# Developments and Challenges Involving Triplet Transfer across Organic/Inorganic Heterojunctions for Singlet Fission and Photon Upconversion

**DOI:** 10.1021/acs.jpclett.3c03013

**Published:** 2023-12-06

**Authors:** Sourav Maiti, Laurens D. A. Siebbeles

**Affiliations:** † Central Laser Facility, RCaH, STFC-Rutherford Appleton Laboratory, Harwell Science and Innovation Campus, Didcot OX11 0QX, United Kingdom; ‡ Chemical Engineering Department, Delft University of Technology, Van der Maasweg 9, 2629 HZ Delft, The Netherlands

## Abstract

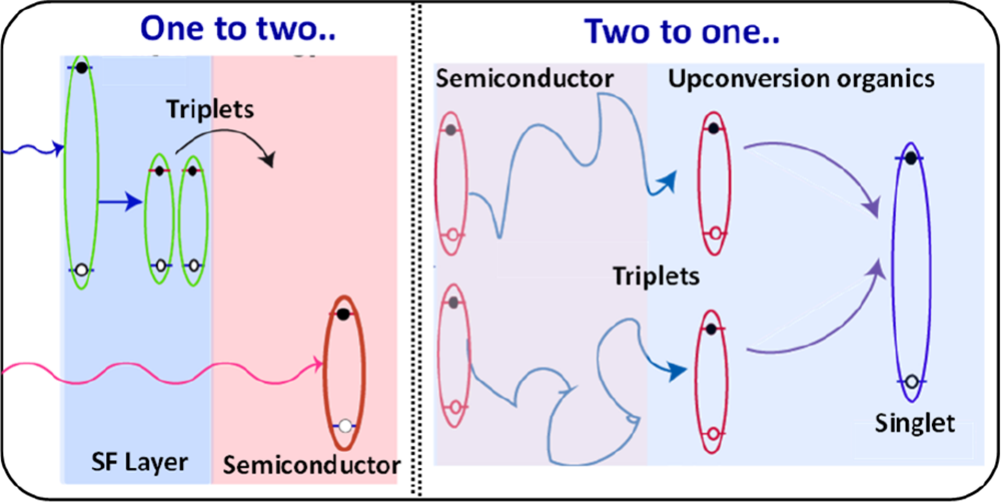

In this Perspective, we provide an overview of recent advances in harvesting triplets for photovoltaic and photon upconversion applications from two angles. In singlet fission-sensitized solar cells, the triplets are harvested through a low band gap semiconductor such as Si. Recent literature has shown how a thin interlayer or orientation of the singlet fission molecule can successfully lead to triplet transfer. On the other hand, the integration of transition metal dichalcogenides (TMDCs) with suitable organic molecules has shown triplet–triplet annihilation upconversion (TTA-UC) of near-infrared photons. We consider the theoretical aspect of the triplet transfer process between a TMDC and organic semiconductors. We discuss possible bottlenecks that can limit the harvesting of energy from triplets and perspectives to overcome these.

In this Perspective, we discuss recent advances and prospects of materials for formation and exploitation of electronic excited states (excitons) with a triplet spin configuration. In common organic materials, triplet excitons are much longer lived than singlets. This is because the transition from the triplet state to the ground singlet state is spin-forbidden; that is, the spin angular momentum of one in the triplet state must change to zero in the singlet ground state. This transition requires a spin–orbit interaction, which is small in organic materials that do not contain heavy atoms with a large nuclear charge.

Long-lived triplet excitons are essential intermediate states needed to increase the efficiency of photovoltaics by the processes of singlet fission and triplet–triplet annihilation upconversion (TTA-UC), which are depicted in Figures 1 and 5, respectively. In photovoltaic devices, photons with energy above the band gap of the light-absorbing semiconductor (such as Si) are harvested, while photons with lower energy are not absorbed. Photons with energy in excess of the band gap produce hot electrons and holes that usually lose their excess energy through thermalization. The loss of this excess energy and the absence of absorption of low energy photons are the major factors that determine the efficiency limit of a single junction solar cell. This so-called Shockley–Queisser limit amounts to 33% for solar cells based on Si.^1^ Triplets can help utilize this unexploited energy through two mechanisms: (1) singlet fission to avoid thermal loss from higher-energy photons^2−6^ and (2) triplet–triplet annihilation upconversion (TTA-UC)^7−12^ to harvest lower-energy near-infrared (NIR) photons by converting them to visible photons. Starting from a 28% efficiency of a base Si solar cell, the exploitation of singlet fission can increase the efficiency to 36%^13^ and photon upconversion to 48%.^14^ Interestingly, TTA-UC also has potential applications in biological imaging and photocatalysis.^15−19^

**Figure 1:**
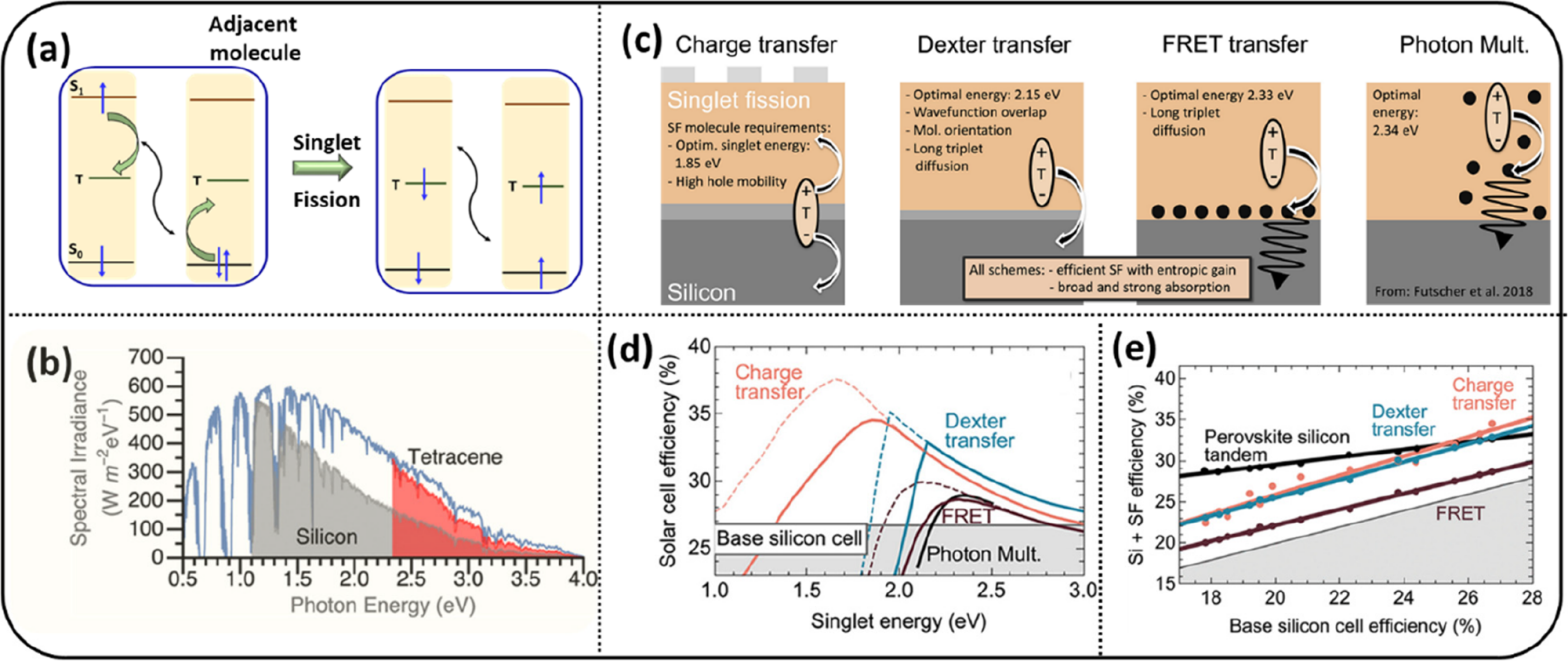
(a) The process of singlet fission in organic semiconductors producing two triplets from one singlet exciton. (b) Absorption of solar light by Si (gray) and theoretical maximum gain in absorption after depositing tetracene on top (red). Reproduced from ref (29). Licensed under a Creative Commons Attribution (CC BY) license. (c) Working principles of singlet fission-sensitized Si solar cells. Low-energy photons pass through the organic singlet fission layer and directly create charge carriers in Si. High-energy photons are absorbed in the organic layer and can each produce two triplets via singlet fission. Different scenarios of harvesting the energy from triplets: charge transfer, Dexter energy transfer, and FRET or photon multiplication via quantum dots. (d) Calculated efficiency of a 28% base Si solar cell for different transfer methods as a function of the singlet energy of the singlet fission material. The solid lines are for 100 meV entropy gain, whereas the dashed line represents an optimistic 300 meV entropy gain. (e) Calculated efficiency as a function of the base efficiency of a Si solar cell and optimal singlet energy for each case. Panels c–e are reproduced from ref (13). Licensed under a Creative Commons Attribution (CC BY NC ND) license.

The utilization of triplet states by heterostructures in a solid-state architecture is an active area of research and a continuously developing field. In this Perspective, we focus on (1) triplet transfer from organic singlet fission materials to Si or a perovskite and (2) energy transfer across semiconductor/organic interfaces followed by TTA-UC with particular attention to transition metal dichalcogenides (TMDCs) as NIR sensitizers.

In singlet fission, absorption of a photon initially leads to formation of a singlet excited state that decays into two triplet excitons (Figure 1a).^2^ Singlet fission has analogy with carrier multiplication^20−22^ in inorganic materials, in the sense that in both processes a single photon excites more than one electron. A difference is that singlet fission specifically produces two triplet states, while in the case of carrier multiplication even more than two electrons can be excited and the electron–hole pairs do not need to form triplet excitons. Figure 1a depicts the usual case of singlet fission in an organic material, where initial photoexcitation produces a Frenkel exciton mainly localized on one molecule, followed by singlet fission, leading to two triplets on adjacent molecules. Oligoacenes such as tetracene, pentacene, and hexacene show efficient singlet fission. After their formation the triplets need to move away from each other to prevent triplet–triplet annihilation. Next, they must move to the interface with a semiconductor material and dissociate into free charges that can contribute to the photocurrent of a solar cell.

Triplet energy transfer from organic molecules to Pb-chalcogenide nanocrystals has been reported for tetracene/PbS^23^ and pentacene/PbSe^24^ heterostructures. Subsequent studies have shown surface anchoring the organic on the QD surface significantly enhances the triplet transfer efficiency.^25,26^ Singlet fission/QD systems have been reviewed recently.^27^ However, for photovoltaic applications, it is essential that highly mobile free charge carriers are produced. To achieve this, the use of a bulk crystalline semiconductor is highly promising. Therefore, we focus on heterostructures of an organic singlet fission layer on top of silicon (Si) or a perovskite.

Enhancing the performance of a Si-based solar cell by exploitation of singlet fission can occur via different processes. Depositing a layer of a singlet fission material on top of Si can in the first place enhance the absorption of solar light, as shown in Figure 1b for a tetracene/Si hybrid solar cell. The triplet energy can in several ways be converted into electrical current as shown in Figure 1c.^28^ In the case of charge transfer, triplets diffuse to the organic/Si interface and electrons are injected into the Si layer, whereas the hole remains in the organic layer. The mechanism via Dexter energy transfer involves the diffusion of triplets to the interface, where they directly transfer their energy to Si. Another pathway involves triplet transfer to quantum dots on top of the Si surface first, followed by Förster resonance energy transfer (FRET) to Si. Alternatively, the quantum dots can be embedded in the organic layer, and after triplet energy transfer to quantum dots, the subsequently emitted light is reabsorbed by Si. Figure 1d shows the calculated (gain in) efficiency for these scenarios for a base 28% efficient Si solar cell as a function of the singlet energy. Figure 1e shows this as a function of the base Si solar cell efficiency and optimal singlet energy.^13^ According to the calculations an efficiency of ∼35% can be achieved for the cases of charge transfer and Dexter energy transfer.^13^

The efficiency of the charge and triplet energy transfer processes in Figure 1c is, to a large extent, limited by quenching at the organic/Si interface. MacQueen et al. studied a photovoltaic device based on a heterojunction of tetracene deposited on top of Si and from simulation they inferred an exciton transfer yield of only 8%.^30^ Einzinger and co-workers showed for the first time that triplet transfer from tetracene to Si can be realized with an ∼8 Å thick hafnium oxynitride interlayer between the two materials (Figure 2a).^31^ Observing the Si photoluminescence upon photoexciting tetracene in the tetracene/interlayer/Si structure strongly suggests the transfer of triplets to Si (Figure 2b). To confirm the triplet transfer process, magnetic field-dependent photoluminescence and photocurrent were measured. In the presence of a magnetic field (>0.03 T), the singlet fission process slows down, leading to a smaller number of triplets.^32^ As the triplet population subsides in a magnetic field, the photocurrent becomes smaller, and the change in photocurrent thus becomes negative. Hence, the negative changes in Figure 2c imply a contribution of charges that result from singlet fission.

**Figure 2:**
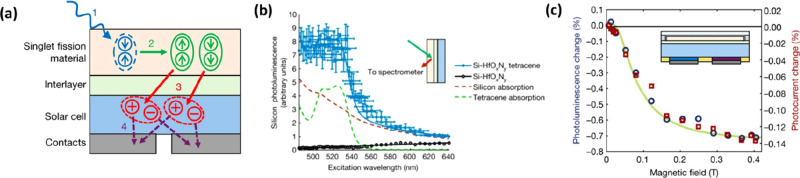
(a) A thin interlayer passivates the Si surface and allows triplets to pass through. (b) Si photoluminescence excitation spectra of the tetracene/interlayer/Si assembly (blue). (c) Magnetic field dependence of Si photoluminescence and photocurrent change of the solar cell. Reproduced with permission from ref (31). Copyright 2019 Springer Nature.

Subsequently, Daiber and Maiti et al. showed that triplet transfer from tetracene to Si can be achieved without an interlayer.^33^ Triplet transfer was probed through magnetic field-dependent photocurrent measurements, as shown in Figure 3a. Surprisingly, after exposure to air, the change in photocurrent from tetracene/Si solar cells became negative, implying triplet transfer from tetracene to Si. A control sample of tetracene/SiO_*x*_/Si did not show any change in the photocurrent upon exposure to air. X-ray diffraction (XRD) of tetracene before and after air exposure showed a change in the morphology of the tetracene (Figure 3b). Tetracene exhibits two different crystal structures known as polymorphs TCI and TCII with slightly different XRD spectra. The change of the XRD spectrum upon air exposure can be attributed to a fraction of the TCI polymorph being converted into TCII and concomitant more efficient singlet fission and triplet transfer to Si. The tetracene photoluminescence in a solid layer shows fast decay due to singlet fission and a long-lived component due to TTA regenerating a fraction of singlets. Thus, tetracene photoluminescence provides both singlet and triplet lifetimes. Figure 3c compares the photoluminescence decays for tetracene/Si and a reference sample tetracene/SiO_*x*_/Si. Due to the presence of the SiO_*x*_ blocking layer, no triplet transfer is possible in the tetracene/SiO_*x*_/Si sample. Upon exposure to air, the triplet decay becomes faster in tetracene/Si compared to tetracene/SiO_*x*_/Si, which we attribute to triplet transfer to Si (Figure 3c). The triplet transfer efficiency was calculated from photoluminescence decay measurements and found to be around 35% (Figure 3d). Apparently, controlling the tetracene morphology has an important effect on triplet transfer. A study by Arias et al. has shown that the singlet fission rate is faster in the TCII polymorph compared to TCI.^34^ These studies signify the importance of crystal packing for both singlet fission and triplet transfer efficiencies. Future studies are needed on the effects of integrating a passivation layer (such as hafnium oxynitride) and realizing the optimal morphology of tetracene to increase the triplet transfer efficiency.

**Figure 3:**
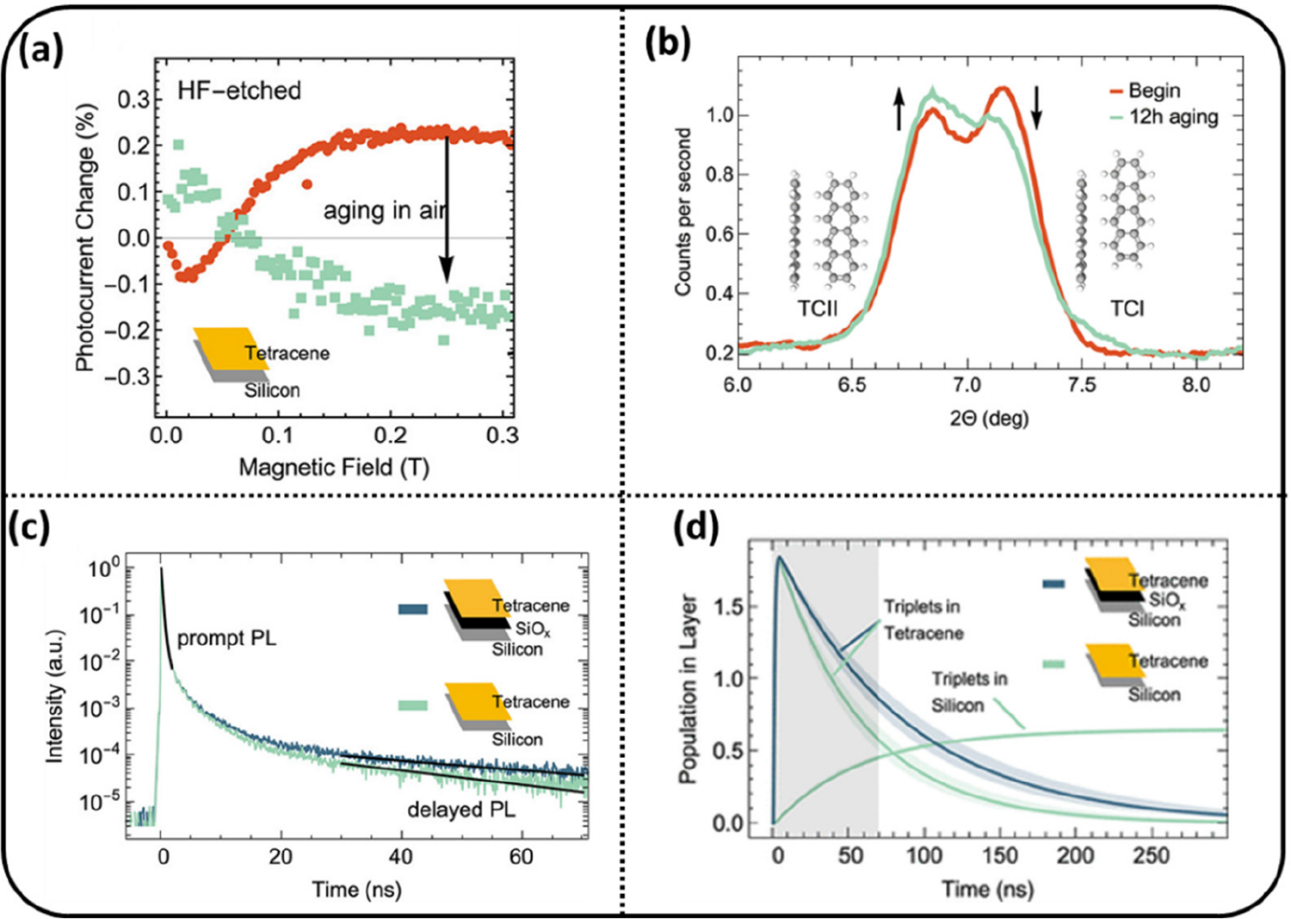
(a) Magnetic field dependence of Si photocurrent. (b) Change in tetracene polymorphism due to air exposure. (c) Decay of tetracene photoluminescence after air exposure showing faster decay of triplets in tetracene/Si compared to the control sample tetracene/SiO_*x*_/Si. (d) Modeling of photoluminescence data shows that ∼35% of triplets transfer to Si. Reproduced from ref (33). Licensed under a Creative Commons Attribution (CC BY NC ND) license.

Triplet transfer from the organic singlet fission layer to an inorganic semiconductor will become more efficient if the organic molecules can be surface anchored to enhance electronic coupling between the molecular orbitals and the electronic Bloch states in the semiconductor. Niederhausen et al. have shown that tetracene is oriented perpendicularly on a Si surface, resulting in poor electronic coupling that limits triplet transfer.^35^ If the orientation of tetracene is parallel to the Si surface, larger electronic coupling can be obtained. More recently, van den Boom et al. successfully managed to covalently bind functionalized tetracene on a Si surface (Figure 4).^29^ Such a seed layer was formed for two derivatives of tetracene with an ethynyl linker at the 2- or 5-position. A layer of normal pentacene with a thickness of 100 nm was deposited on top of the seed layer. Interestingly, the position of the ethynyl linker on the seed layer tetracene molecules has a significant effect on the orientation of the nonfunctionalized tetracene molecules in the layer that is deposited on top. XRD measurements showed that a seed layer with 2-ethynyltetracene drives the tetracene molecules in the top layer to attain the TCII orientation, which is favorable for triplet transfer.^34^ This means that 2-ethynyltetracene in the seed layer acts to orient the tetracene molecules in the layer deposited on top of it. However, this effect of orienting tetracene mainly occurs close to the seed layer and did not significantly increase the level of triplet transfer to Si, as inferred from magnetic field-dependent photocurrent measurements. However, this study shows promise that tetracene and probably other singlet fission molecules can be favorably oriented on a Si surface.

**Figure 4:**
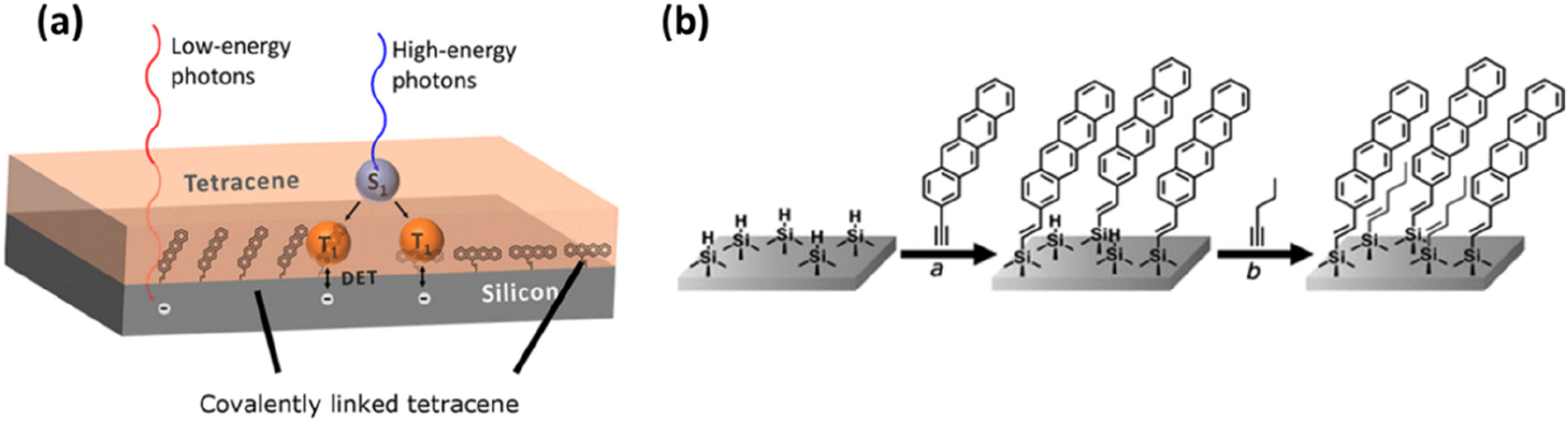
(a) Schematic of covalently linked tetracene on a Si surface. (b) The hydrogen-terminated Si reacted with tetracene linkers (2-ethynyltetracene is shown here), leading to covalent attachment. The surface is filled with 1-pentyne to protect the unreacted Si–H surface from oxidation. Reproduced from ref (29). Licensed under a Creative Commons Attribution (CC BY) license.

Perovskites currently attract a great deal of attention for solar cell applications. Low band gap perovskites are of interest for singlet fission-sensitized perovskite solar cells. Bowmann et al. investigated tetracene and 1,6-diphenyl-1,3,5-hexatriene as singlet fission sensitizing molecules in halide perovskite solar cells through magnetic field-dependent photoluminescence spectroscopy.^36^ Transfer of triplets from tetracene to the perovskite was not observed. Theoretical modeling of the interface revealed weak interaction between tetracene and the perovskite, resulting in triplets remaining localized in the tetracene layer. The tetracene molecules were found to be oriented perpendicular to the perovskite surface. This orientation leads to small electronic coupling and poor triplet transfer, as in the case for Si discussed above. TMDCs can also be used as potential sensitizers with singlet-fission organics.^5^ Jang et al. demonstrated electron transfer from the triplet states in pentacene (triplet energy 0.86 eV) to MoTe_2_ (band gap 1.1 eV) doubling the photocurrent in a bilayer device.^37^ This type of bilayer solar cells are beneficial for exceeding the Shockley–Queisser limit as the MoTe_2_ shows efficient carrier multiplication (QY ≈ 2 for 2*E*_g_ < *E* < 3*E*_g_, where *E*_g_ is the band gap). Ye et al. have shown for TIPS-pentacene/MoS_2_ heterostructure triplets generated by singlet fission dissociate at the interface and transfer electrons to MoS_2_ enhancing the photocurrent beyond 100%.^38^ Low band gap Sn/Pb halide perovskites also show efficient carrier multiplication^39−41^ and photocurrent enhancement^40,41^ at more than twice the band gap. Therefore, we envisage that a low band gap perovskite/singlet fission organic combination will be impactful for future devices.

We conclude that the morphology of the organic singlet fission layer and the orientation of the molecules that are covalently bound to the semiconductor in the seed layer need to be controlled to realize efficient singlet fission and triplet transfer to the semiconductor. Theoretical modeling of electronic coupling between singlet fission molecules and the semiconductor can play an important role in finding suitable molecules with appropriate surface orientation. Recently, reports of the computational “inverse design” method have appeared, which allow one to predict a material structure with desired properties.^42,43^ This method has been used to find the tetracene structure that is optimal for singlet fission.^43^ Finding the orientation of singlet fission molecules on the surface of a semiconductor that optimizes interfacial triplet transfer may also benefit from the use of the inverse design method.

Singlet fission may be considered as a down-conversion process in which a visible photon finally produces two electron–hole pairs, with each having energy close to half of the photon energy. The opposite process of triplet–triplet annihilation upconversion (TTA-UC) is also of interest to convert the solar energy spectrum more effectively to electrical energy. Through TTA-UC, two electron–hole pairs generated by two photons of lower energy are converted into one photon with about twice the energy. For TTA-UC the key components are a sensitizer and an annihilator (Figure 5a). The sensitizer absorbs in the NIR region using materials such as two-dimensional (2D) transition metal dichalcogenides (TMDCs) or Pb-chalcogenide NCs. The sequential (or simultaneous) injection (process 1) of electrons (e^–^) and holes (h^+^) leads to formation of triplets in the annihilator. The triplets then need to undergo bimolecular triplet–triplet annihilation (process 2) to form excited singlet states that emit a photon (process 3) to be absorbed in a solar cell (Figure 5b). The last process occurs in competition with unwanted back energy transfer, where the singlet states transfer their energy back to the sensitizer (process 4). In some cases, there is a separate emitter component which accepts the singlet from the annihilator. Significant progress has been achieved with perovskites and semiconducting nanocrystals as sensitizers for TTA-UC.^44−49^ In addition, TMDCs have great prospects for use as a triplet sensitizer, as we discuss below.^50−56^

**Figure 5:**
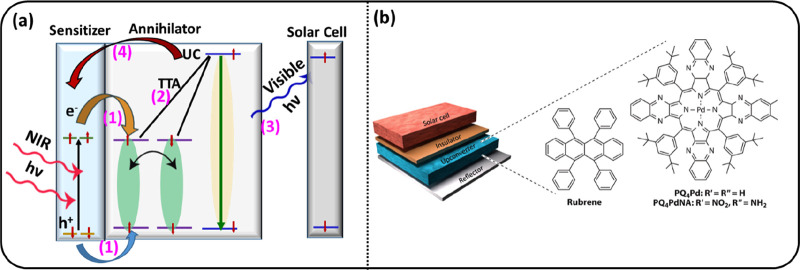
(a) Triplet–triplet annihilation upconversion (TTA-UC) transforms near-infrared light into visible photons. Processes involved in TTA-UC. (b) A representative solar cell device with an upconversion layer. In this example, a Pd-based complex is used as sensitizer. Reproduced from ref (7). Licensed under a Creative Commons Attribution (CC BY) license.

Before addressing upconversion of light, we first address issues involving charge and energy transfer between TMDC sensitizers and organic molecular upconverter layers (see Figure 6). TMDCs are of interest due to strong light absorption, absence of surface ligands, and the possibility to control the number of stacked monolayers. The latter enables tuning of electronic band structure and exciton properties.^57,58^ Depending on the band alignment of the TMDC and organic material, the excitons initially photogenerated in the TMDC layer can decay by charge transfer,^50−54,59^ singlet exciton transfer,^55^ or triplet exciton transfer.^56^ Representative examples of charge transfer are between tetracene/WS_2_ heterointerface (Figure 6a),^51^ pentacene/MoS_2_ heterointerface (Figure 6b),^52^ and anthracene-based organics/MoS_2_ heterointerface (Figure 6c),^53^ whereas singlet transfer occurs in the tetracene/WSe_2_ heterointerface (Figure 6d).^55^ Here we are going to focus on the transfer of triplets from the organic to the TMDC (Figure 6e,f). Kafle et al. demonstrated that photoexcitation of zinc-phthalocyanine (ZnPc) on molybdenum disulfide (MoS_2_) (Figure 6a) leads to electron injection from the ZnPc singlet state into MoS_2_, followed by electron back-transfer producing triplets in the ZnPc layer.^50^ Maiti et al. performed a transient optical absorption study on a rhenium disulfide (ReS_2_)-tetracene heterostructure. They found that photoexcitation of ReS_2_ leads to formation of triplet excitons in the tetracene layer (Figure 6f).^56^ The tetracene triplet energy (1.2 eV) is smaller than the ReS_2_ band gap, allowing this process to be energetically allowed. In agreement with this, the population of singlets in tetracene is not affected by the photoexcitation of ReS_2_ since the energy of tetracene singlets is much higher than the ReS_2_ band gap. The formation of triplets in tetracene occurs within 5 ps, which is much faster than for Pb-chalcogenide nanocrystal or perovskite sensitizers for which triplet formation on the time scale of tens to hundreds of nanoseconds has been reported.^60−62^ The faster triplet formation in tetracene deposited onto ReS_2_ is likely due to the direct contact between the two materials, while Pb-chalcogenide nanocrystals are passivated with large surface ligands that hinder electronic coupling to other molecules.

**Figure 6:**
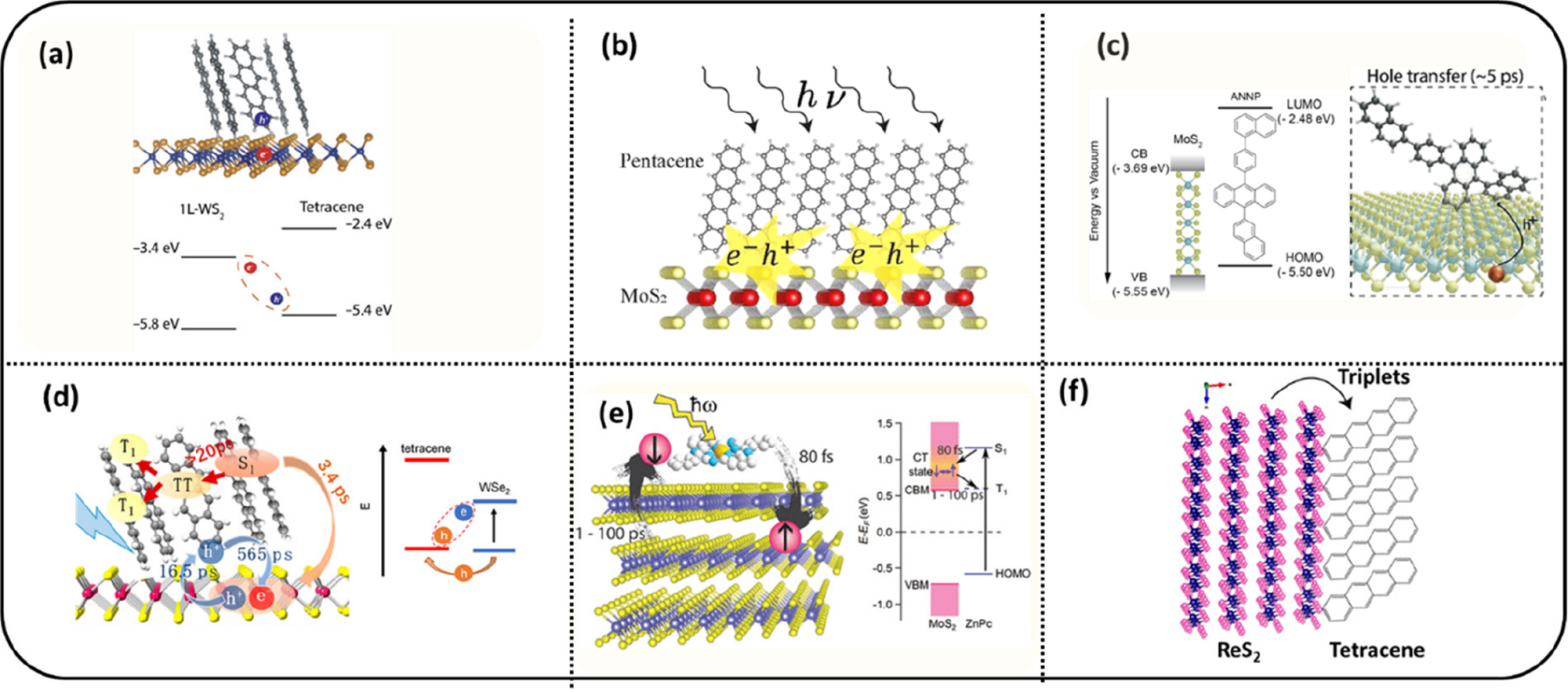
Examples of charge or exciton transfer in organic/TMDC bilayers. (a) Formation of charge transfer state at the tetracene/WS_2_ interface.^51^ Reproduced from ref (51). Licensed under a Creative Commons Attribution (CC BY NC) license. (b) Hole transfer from MoS_2_ to pentacene in MoS_2_/pentacene bilayer deposited on Au surface.^52^ Reproduced with permission from ref (52). Copyright 2022 American Chemical Society. (c) Charge separated state through hole transfer from TMDC to organic in the MoS_2_/ANNP interface. Trapped excitons are formed when both TMDC and organic are excited at 400 nm.^53^ Reproduced with permission from ref (53). Copyright 2023 American Chemical Society. (d) Singlet transfer together with hole transfer and charge-separated state in WSe2/tetracene.^55^ Reproduced with permission from ref (55). Copyright 2021 American Chemical Society. (e) Charge separation and subsequent triplet formation in a zinc-phthalocyanine/MoS_2_ heterostructure.^50^ Reproduced with permission from ref (50). Copyright 2017 American Chemical Society. (f) Triplet generation in tetracene upon photoexcitation of ReS_2_.^56^ Reproduced from ref (56). Licensed under a Creative Commons Attribution (CC BY NC ND) license.

Recently, Duan et al. reported TTA-UC from an organic layer upon photoexcitation of a TMDC layer.^63^ The design involved a monolayer of MoSe_2_ as an NIR sensitizer and a layer of rubrene as annihilator. The rubrene layer contains a small amount of dibenzotetraphenylperiflanthene (DBP) as a visible emitter (Figure 7a). Photoexcitation of MoSe_2_ at 772 nm resulted in upconverted emission from DBP near 610 nm (Figure 7b). The proposed mechanism involves the generation of excitons in the TMDC layer followed by interfacial triplet energy transfer to rubrene. The subsequent TTA in rubrene produces singlets, which are transferred to DBP through FRET and subsequently emit upconverted photoluminescence. The MoSe_2_ photoluminescence decay in Figure 7c is faster in the presence of rubrene due to exciton transfer to rubrene within 20 ps. The time-resolved upconverted photoluminescence from DBP provides information about the mechanism of the TTA process. The upconverted emission in Figure 7d shows growth components of ∼14 and ∼261 ns, which are attributed to fast TTA of triplets at high concentration near the MoSe_2_/rubrene interface and of triplets at lower concentration after their diffusion away from the interface. Eventually the upconverted photoluminescence decays on a microsecond time scale, which is determined by the triplet lifetime. The upconversion quantum yield was found to be ∼1.1% for 772 nm photoexcitation with 100 W/cm^2^ power, which is higher than results for PbS nanocrystals or perovskite-based systems.^63^ Multiple MoSe_2_ monolayers were stacked to enhance the optical absorption. Unfortunately, the upconversion yield decreased significantly. The decrease is due to slow triplet diffusion between the stacked monolayers that are coupled by weak van der Waals interactions, which must compete with exciton relaxation. The exact mechanism of triplet formation in the organic layer still remains a question. The electron–hole pair in the TMDC layer can transfer simultaneously (exciton transfer mechanism) or sequentially (charge transfer mechanism) to the organic layer.

**Figure 7:**
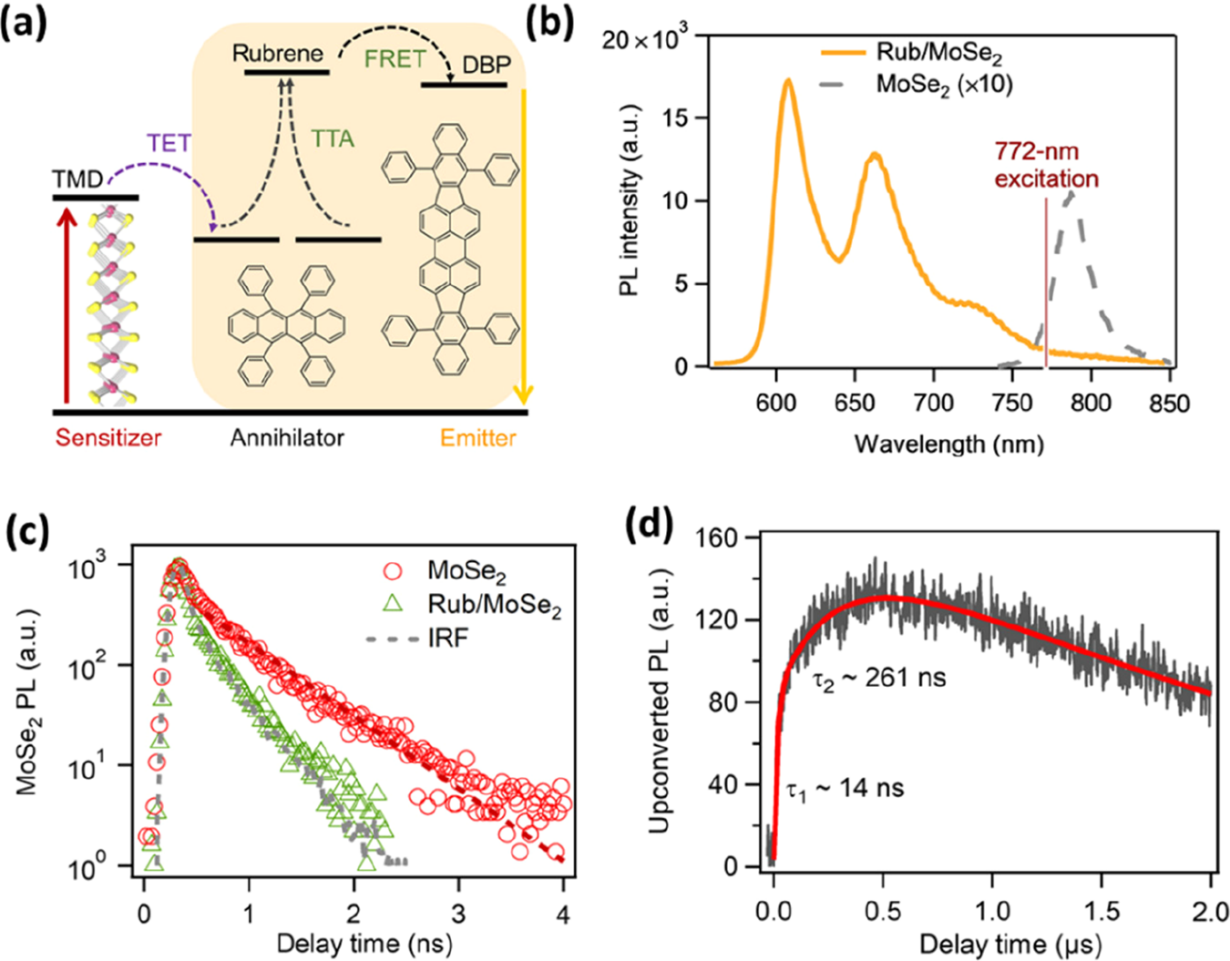
(a) The TTA-UC system here consists of a sensitizing MoSe_2_ monolayer and an annihilation layer of rubrene, which contains DBP molecules as emitters. (b) Upconverted emission from DBP after photoexcitation of MoSe_2_. (c) Photoluminescence from MoSe_2_ decays faster in the presence of rubrene/DBP due to exciton transfer from MoSe_2_ to the latter. (d) Upconverted photoluminescence decay from DBP. Reproduced from ref (63). Licensed under a Creative Commons Attribution (CC BY NC) license.

Analogous to the study above on MoSe_2_ as sensitizer, Dziobek-Garrett et al. have shown upconversion from a heterostructure of a WSe_2_ monolayer as sensitizer and a layer of rubrene containing DBP molecules as emitters (Figure 8a).^64^ The upconverted emission (Figure 8c) was visible to the naked eye (Figure 8d). The exciton energy level diagram in Figure 8b shows there is considerable energy loss during the conversion of the exciton (X) in WSe_2_ with energy of 1.6 eV to the triplet in rubrene with energy of 1.1 eV. There is a need for studies to reduce this loss by using other combinations of 2D and organic materials.

**Figure 8:**
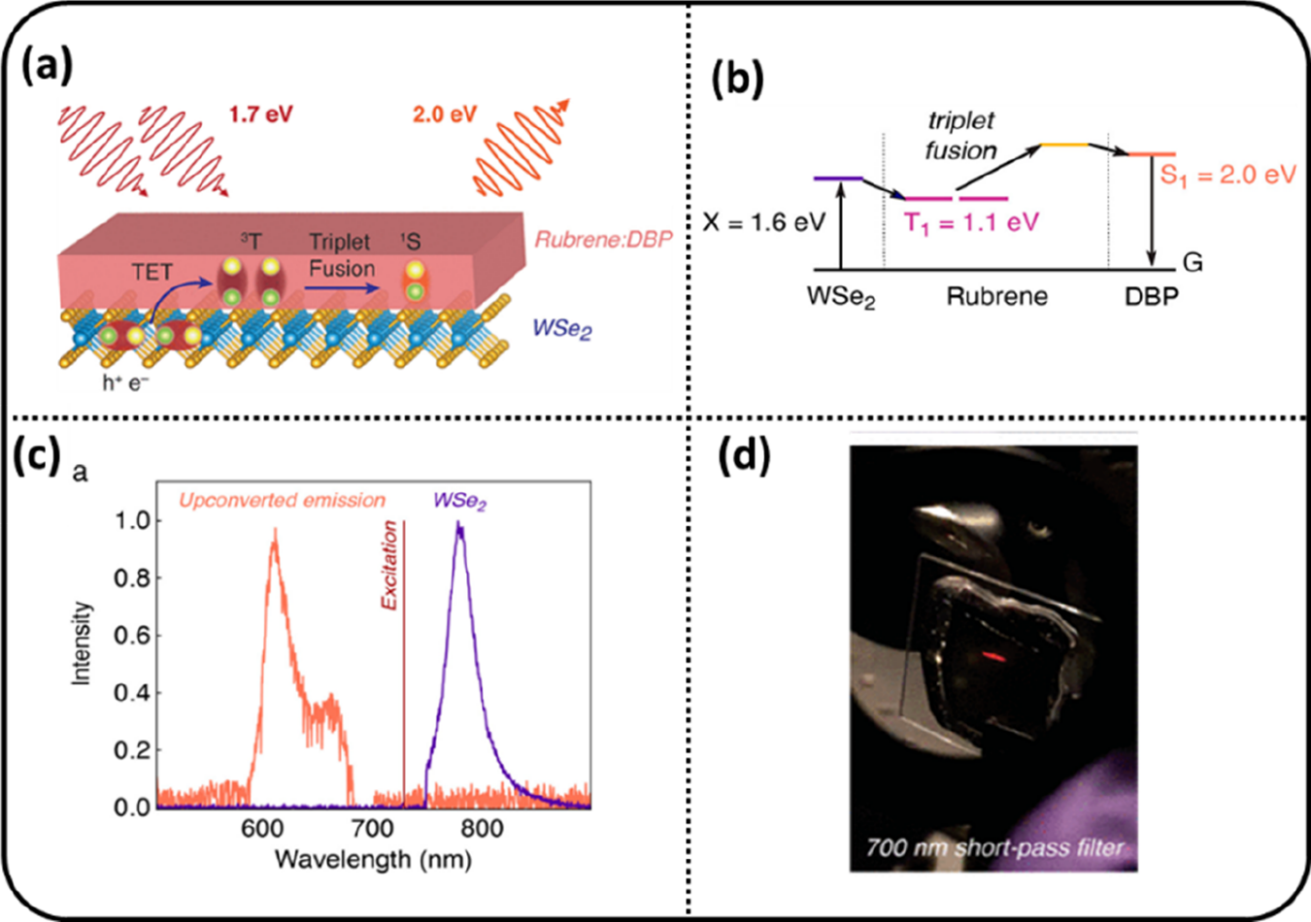
(a) Schematic of photon upconversion in a WSe_2_ monolayer as a sensitizer and an annihilation layer of rubrene containing DBP as an emitter. (b) Exciton energy level diagram. (c) Upconverted emission from DBP. (d) The upconverted emission is visible through a camera on a mm^2^ scale. Reproduced with permission from ref (64). Copyright 2023 American Chemical Society.

Very recently de Clercq et al. demonstrated photogeneration of self-trapped excitons in a heterostructure of an MoS_2_ monolayer onto which a layer of 9-(2-naphthyl)-10-[4-(1-naphthyl)phenyl]-anthracene (ANNP) was deposited by thermal evaporation.^53^ The traps were assigned to structural deformations in the ANNP and could be removed by annealing. From analysis of optical pump–probe spectroscopy data it could be inferred that self-trapped excitons in ANNP are formed away from the interface with MoS_2_. Interestingly, a layer of ANNP on quartz did not exhibit self-trapped excitons. This leads to the conclusion that the interfacial interaction between the ANNP molecules and MoS_2_ has a significant long-range effect on the crystal structure of ANNP.

From the encouraging results of TTA-UC described above, we conclude that TMDCs show great promise to produce triplets in an adjacent organic layer. Important challenges include optimization of the orientation of molecules on the TMDC surface, the use of more efficient upconverting molecules with minimal energy loss, and the fabrication of defect-free TMDC/organic heterojunctions.

In conclusion, we have discussed recent progress in harvesting long-lived triplets through singlet fission-sensitized Si or a perovskite and photon upconversion using TMDCs. For both singlet fission and TTA-UC it is an important challenge to enhance the efficiency of exciton transfer between the organic and inorganic materials. This requires a strong electronic coupling between them. It is essential to realize a favorable orientation for strong coupling between molecules at the organic/inorganic interface. Interestingly, the interfacial orientation may also affect the molecular packing further away from the interface and, in turn, the efficiency of diffusion of triplets. To make effective use of singlet fission, diffusion of triplets to the inorganic layer must be fast so that it can compete with their decay. For TTA-UC, fast triplet diffusion is important to realize efficient singlet formation by bimolecular triplet–triplet annihilation.
